# T-cell repertoire diversity: friend or foe for protective antitumor response?

**DOI:** 10.1186/s13046-022-02566-0

**Published:** 2022-12-22

**Authors:** Nicla Porciello, Ornella Franzese, Lorenzo D’Ambrosio, Belinda Palermo, Paola Nisticò

**Affiliations:** 1 Tumor Immunology and Immunotherapy Unit, IRCCS-Regina Elena National Cancer Institute, Rome, Italy; 2 Department of Systems Medicine, University of Rome Tor Vergata, Via Montpellier 1, 00133 Rome, Italy

**Keywords:** T-Cell Receptor, Repertoire, Diversity, Clonality, Immune checkpoint inhibitors, Cancer vaccination, TCR-seq, Single-cell, Biomarker, Cancer

## Abstract

Profiling the T-Cell Receptor (TCR) repertoire is establishing as a potent approach to investigate autologous and treatment-induced antitumor immune response. Technical and computational breakthroughs, including high throughput next-generation sequencing (NGS) approaches and spatial transcriptomics, are providing unprecedented insight into the mechanisms underlying antitumor immunity. A precise spatiotemporal variation of T-cell repertoire, which dynamically mirrors the functional state of the evolving host-cancer interaction, allows the tracking of the T-cell populations at play, and may identify the key cells responsible for tumor eradication, the evaluation of minimal residual disease and the identification of biomarkers of response to immunotherapy. In this review we will discuss the relationship between global metrics characterizing the TCR repertoire such as T-cell clonality and diversity and the resultant functional responses. In particular, we will explore how specific TCR repertoires in cancer patients can be predictive of prognosis or response to therapy and in particular how a given TCR re-arrangement, following immunotherapy, can predict a specific clinical outcome. Finally, we will examine current improvements in terms of T-cell sequencing, discussing advantages and challenges of current methodologies.

## Background

The advent of immunotherapy, including vaccines, Immune Checkpoint Inhibitors (ICIs), adoptive T-cell and Chimeric Antigen Receptor-T (CAR-T) cell therapy, buttressed by the development of accurate experimental and computational tools, has revolutionized the field of anti-cancer treatment, thus instigating the need of a deep characterization in terms of T-cell clonality, antitumor functionality and clinical effectiveness of immunotherapy.

T lymphocytes are the central mediators of the adaptive immune system and play a crucial role in immune surveillance and cancer eradication. T cells clonally display on their cell surface an enormous number of different antigen (Ag)-specific TCRs which are multimeric complexes, made of a heterodimer of α and β chains, forming the Ag-binding site (TCR), and the signaling-transducing subunits (CD3) [1–3]. Each TCR can recognize short peptides derived from the degradation of self and foreign proteins, presented by major histocompatibility complex (MHC) [4]. Specific TCR-pMHC interaction stimulates thymocyte development into immunocompetent T cells, triggers clonal expansion and differentiation into mature and phenotypically distinct T cells in the periphery, while preserving self-tolerance. Effective T-cell responses rely on highly diverse TCR repertoires which ensure the capability to identify a wide range of Ags [5]. Three stages, mostly occurring in the thymus, shape TCR diversity: somatic recombination of variable (V), diversity (D, β chain only), and joining (J) gene segments to generate variable TCR α and β chains, followed by random nucleotide insertion or deletion between the spliced gene V(D)J junctions [6], and combinatorial pairing which ultimately may lead to a theoretical generation of up to 2 × 10^19^ single αβ chain pairs [7]. However, although extraordinary, TCR diversity is limited and constantly shaped by both thymic production and, in the periphery, by homeostatic mechanisms keeping in check cell proliferation and death. Diversity is further reduced by natural selective pressures [8–10], and even more so by the aging process, as senescence gradually impairs the potential of naïve T cells to respond to new Ags leading to higher representation of more differentiated effector and/or memory T-cell pools [11]. The development of major technical and computational advancements allowed to uncover more layers of complexity underlying T-cell diversity, leading to the introduction of two novel concepts: richness, that refers to the number of unique elements in a population, and evenness, referring to the distribution of the frequencies of those elements [12]. The introduction of the latter parameter contributed to the distinction between different clonal compositions, as richness alone does not fully describe cell expansion among the limitedly distributed T-cell populations [13]. Repertoire diversity is inversely related to clonality, which refers to the number and frequency of observed TCRs within a sample, and together with diversity is often used as a meter of immune response efficacy (please see Glossary section for definitions and quantification of repertoire diversity measures). Thus, the analyses of the TCR repertoire, considering its crucial role in Ag recognition and T-cell activation, can provide precise information on T-cell development, expansion and differentiation [14]. When combined with the evaluation of the immune phenotype and functional features, TCR sequencing represents an outstanding tool for the identification and monitoring of longitudinal and/or treatment-driven Ag-specific T-cell dynamics [15–19].

### TCR profiling as a proxy to uncover quality and quantity of the antitumor immune response

The possibility to deeply investigate the TCR repertoire, also in relation with biological responses, has led to a paradigm shift in the field, particularly at the urging of the new high throughput NGS-based and single-cell approaches [20–23]. Pioneering studies of TCR profiling not only provided a more accurate evaluation of TCRαβ diversity, but they also revealed the contribution of specific TCRβ genes to the onset of certain diseases [24–26], and informed on how T-cell clonality, diversity and specificity, shape biological functions and induce profound impact either on infections, immune-mediated diseases, and cancer evolution. With the development of immunotherapeutic approaches [27], the question of how the cancer genome and the TCR repertoire impact each other, editing the tumor from the early to late stages, has recently come to great attention. In particular, TCR-sequencing (seq) combined with single-cell (sc) RNA-seq has allowed to investigate gene expression, clonal expansion, TCR lineage and Ag specificity of T cells at the tumor site and its adjacent tissues. For instance, despite recent progress of CAR-T and TCR-based adoptive cell therapies, durable clinical success remains challenging [28]. Specifically, TCR-based approaches should allow to target, more efficiently, a broader range of Tumor Antigens (TAs) including extracellular and intracellular epitopes which are often shared among patients [29]. However, the dependency on specific human leukocyte antigen (HLA) alleles, the occurrence of on-target off-tumor toxicity, and the occurrence of primary or acquired and T cell‐intrinsic or extrinsic resistance make only few patients able to benefit from these approaches [30, 31]. Hence, the identification of a larger number of TCRs specific for either new or already identified TAs would be of high clinical relevance. Moreover, even if these naturally selected TA-specific T cells are enriched at the tumor site, they often become dysfunctional by a suppressive Tumor Microenvironment (TME) [32]. Functional TCR profiling showed that absence of tumor reactivity is either explained by T-cell exhaustion, and paucity of tumor reactive intratumoral CD8^+^ T cells [33], highlighting the importance of developing approaches that increase the quality and quantity of the intratumoral TCR repertoire in combination with clinical efforts to reactivate exhausted T cells. Moreover, TA-specific Tumor Infiltrating Lymphocytes (TILs) might acquire transcriptional trajectories and proliferation rates distinct from bystander T cells because of a chronic stimulation, leading to the identification of biomarkers distinguishing tumor-reactive T cells from bystander T cells, providing more effective and safer immune-based therapies [34, 35].

Genetic and epigenetic alterations across tumor progression result in tumor heterogeneity, a well-recognized determinant of tumor outcome [36]. Likewise, the antitumor immune response co-evolves with the tumor, giving rise to an enormous heterogeneity of responses. The observed intra- and inter-individual heterogeneity arouses an important question on the contribution of intrinsic differences in TCR repertoire and its role in patient clinical outcome. This hypothesis has been elegantly supported by Woolaver RA and colleagues by coupling single-cell transcriptomic analysis with TCR profiling, in a head and neck squamous cell carcinoma mouse model. They have shown that genetically identical hosts responded heterogeneously to the same tumor, as revealed by the usage of different TCRs against the same Ags [37]. The authors also found that TILs from both progressor and non-progressor hosts underwent clonal expansion. Top TCR clonotypes and TCR specificity clusters were mutually exclusive between progressors vs. non-progressors, revealing that intrinsic differences in TCR repertoire and different transcriptional trajectories of TILs from different hosts may explain the observed heterogeneous antitumor immune response [37]. However, the few TCR clonotypes shared among the two groups showed diverse functionality, suggesting that beside the TCR itself, signals from the TME also impact on T-cell activation and response.

The extent of tumor T-cell infiltration is a highly informative prognostic factor for many cancer types [38–41], thus it would be relevant to expand our understanding of TME complexity to untangle qualitative and quantitative aspects of antitumor responses for more effective therapeutic opportunities [33]. Lastly, the impact of specific spatial distribution and phenotypic states of CD8^+^ T-cell clonotypes within the TME has just commenced to emerge, suggesting yet another level of complexity [42, 43]. By employing spatial TCR sequencing, a study on brain metastasis (BrM) suggested that CD8^+^ T-cell clones infiltrate the TME in an Ag-independent manner, yet Ag-reactive and bystander clones segregate into different niches and develop different functions according to the surrounding stimuli [42]. These findings instigate the possibility to target locally the unique functional niches populated by BrM-infiltrating CD8^+^ T cells with less detrimental and more long-lasting effects on disease control. Recently, Liu et al., developed Slide-TCR-seq, a new tool for deciphering spatial and transcriptomic features of T cell clones, suitable for thoroughly profile T cells in normal and cancer tissues. Interestingly, the characterization of specimens from a patient with clear renal cell carcinoma who developed resistance following PD-1 treatment, allowed to identify high spatial heterogeneity between clonotypes across different tissues (inter-clonotype heterogeneity) and within the same tissue (intra-clonotype heterogeneity). Expression profile of adjacent tumor and immune cells were highly different depending on the adjacent T cell clone, thus revealing a complex relationship between clonality, localization, and gene expression [43].

### Dynamics of TCR repertoire diversity and clonality during antitumoral immune response

Tumor heterogeneity and antitumoral immune response are dynamically related, as mutations, by generating neo-Ags may elicit a specific host antitumor immune response, concomitant with a potent selective immune pressure driving immune/tumor evolution [44, 45]. A positive relationship between clonal (present in all tumor cells) neoantigen burden and improved T-cell infiltration, patient survival and response to immunotherapy has been recently described in Non-Small Cell Lung Cancer (NSCLC) patients. This suggests that neoantigen heterogeneity may influence antitumor response, thus supporting the development of therapeutic strategies targeting clonal neoantigens. [46]. Moreover, different TCR clones occupy distinct niches at the tumor site, suggesting that intratumoral TCR diversity spatially correlates with genomic heterogeneity [47–49]. Hence, deciphering the TCR repertoire, especially in the context of a more accurate and space-resolved molecular landscape, can help to define the evolution and heterogeneity of the immune response against cancer within an evolving TME.

### A lesson from anti-viral immune response

Considering the striking parallelism between immune responses against chronic viral infection and cancer, the knowledge of the relationship between viral pathogenesis and TCR variability may hint fundamental insights into T-cell-mediated antitumoral immunity. Mechanisms involved in the immune response against viruses include control of pathogen escape and the selection of highly performing Ag-specific cytotoxic T-cell clones often characterized by high affinity and polyfunctionality [50]. Several viruses have developed strategies capable of limiting the magnitude and quality of CD8^+^-mediated response, including mutations that involve residues interacting with the TCR-binding site or impairing Ag presentation by interfering with the MHC-binding groove. Consequently, a narrow TCR repertoire is particularly beneficial to the virus because its cross-reactivity potential is reduced and becomes less adaptable to viral variations, allowing the virus to select the mutations that are most self-beneficial. Narrow TCR repertoires have been described for human chronic viral infections, such as those caused by Epstein-Barr virus, cytomegalovirus, hepatitis C virus and HIV, possibly resulting from the clonal proliferation of T cells constantly exposed to viral Ags [51]. Therefore, learning from antiviral responses, a question arises as to whether mutations enhancing tumor growth may benefit from a narrow TCR repertoire or increase TCR clonality, thus eliciting a more specific antitumor response.

The influence of TCR diversity on the recognition of pMHC complexes has been described in a number of studies [52], although the relationship between TCR diversity and T-cell functionality has not been completely clarified, either in the context of spontaneous or vaccine-induced Ag-specific immune responses. Noteworthy, TCR repertoire diversity is a critical determinant which characterizes T-cell responses both at the periphery and at the tumor site. Presence of T-cell infiltration has been associated with a favorable prognosis in different cancer types [53], however deciphering the functional states of antitumor immune responses may provide major breakthroughs in optimizing cancer therapy. Recently, an elegant study analyzing the intrinsic tumor reactivity of the intratumoral TCR repertoire in ovarian and colorectal cancer (CRC) [33] has found that the competence to recognize autologous Tumor Associated Antigens (TAAs) is limited to nearly 10% of intratumoral CD8^+^ T cells. This observation implies a low intrinsic antitumor capability of TILs, proving the requirement for therapeutic approaches able to increase both quantity and quality of infiltrating T cells.

### Diversity or clonality, that is the question

Investigations addressing the role of TCR clonality/diversity in cancer onset and progression led to different and often contradictory conclusions. Opposite results from different studies can be explained according to different sources of T cells employed for data generation i.e., peripheral vs. tumor site and different methodology i.e., bulk vs. single cell-based approaches. The evaluation of TCR diversity can also be affected by tissue-specific variables, like the different amount of tumor tissue-resident memory T cells, endowed with higher clonality [54], across different tissues. First studies on TCR repertoire profiling were obtained in melanoma patients where tissue sampling is more accessible and less invasive. A seminal investigation on five metastatic melanoma patients showed the occurrence of highly homologous CDR3 expanded T cells in all analyzed patients, which speaks in favor of a response directed toward a restricted number of epitopes and the usage of a highly restricted TCR repertoire [55]. More recently, by combining high-throughput scRNA-seq and scTCR-seq Oliveira and colleagues investigated the relationship between phenotypic properties and TCR repertoire clonality of melanoma-infiltrating T cells [56]. Their findings depict a scenario where highly expanded clonotypes display an exhausted phenotype, with decreased TCR diversity. The same clonotypes were also found in the periphery, but with a non-exhausted memory phenotype suggesting that the exhausted signature was achieved within the TME [56]. Initial investigations in CRC, showed that peripheral blood from CRC patients have a higher TCR repertoire restriction than healthy donors, that was instead similar in tumor tissue and normal mucosa, suggesting a TCR distribution more compartment-specific rather than due to tumor/T-cell interaction [57]. Conversely, Sherwood et al., described a substantial difference in the TCR repertoire diversity between T cells infiltrating the tumor site and T cells of the healthy mucosa both characterized by a higher TCR clonality compared to that observed in peripheral blood [58]. A highly polyclonal TCR repertoire was shown to characterize T cells infiltrating renal carcinoma, a tumor type that can display extensive genetic intra-tumor heterogeneity [48]. Successively, by using high-throughput TCRβ sequencing and scRNA-seq on both circulating and tumor-infiltrating T cells, it was found that a higher TCR diversity correlates with a more naïve T-cell repertoire and associates to a better prognosis in this tumor [59]. Differently, in a study on ovarian cancer patients, TILs displayed a strong TCR repertoire similarity throughout the tumor site, but different from the circulating counterpart, suggesting that in ovarian cancer, antitumor immune response is spatially homogeneous, but distinct from the periphery [60]. Pancreatic cancer is one of the most lethal tumors where the presence of an abundant desmoplastic stroma promoting malignancy of cancer cells and forming a barrier to immune cell infiltration and activation, is a major driver of cancer aggressiveness [61]. Thus, characterization of the molecular and cellular determinants regulating T-cell enrichment and differentiation in such a peculiar and spatially-complex compartment, would be of critical importance to overcome treatment failure. One of the first studies aimed at profiling the TCR repertoire of infiltrating and circulating cells in pancreatic cancer patients was performed by Bai et al., who found no difference in the periphery of patients and healthy donor repertoire but identified an abundant presence of clones with low frequencies in cancer-infiltrating T cells compared to controls, suggesting the existence of shared immunological features among patients [62]. The homogeneity of intratumor TCR repertoires in pancreatic cancer has been recently confirmed by a study showing high intra-tumoral TCR β repertoire overlap across the tumor, although distinct from the circulating repertoire [63]. In NSCLC, mutational burden and tumor heterogeneity result in highly diverse intratumoral TCR repertoire between samples of different patients, however, T-cell clone overlap analyses between normal lung tissues and NSCLCs showed a good degree of similarity [64] suggesting that several infiltrating clonotypes likely reflect a persistent exposure to pathogens more than a response to specific tumor-Ag. Reuben et al. analyzed the TCR repertoire in 45 tumor regions from 11 localized lung adenocarcinoma samples, confirming a substantial intratumor heterogeneity in T-cell clonality [49]. TCR diversity positively correlated with predicted neo-Ags, suggesting that the different expression of neo-Ags in different tumor regions may be responsible for the longitudinal differences observed in the spatial TCR distribution. Finally, a higher extent of TCR heterogeneity was related to an increased risk of postsurgical relapse and reduced disease-free survival, implying a possible clinical inference for TCR heterogeneity as a potential biomarker in NSCLC prognosis [49]. These findings have been further confirmed by Joshi et al., that in the course of the prospective lung TRACERx study observed a higher chance of recurrence or reduced patient overall survival (OS) in association with intratumoral genomic heterogeneity [65]. Authors, following the hypothesis that mutational load and genomic heterogeneity reflect the intra-tumor TCR repertoire variability, observed that the TCR repertoire across multi-region tumor specimens is different from that observed in normal lung tissue and periphery [65]. Similarly, characterization of T cells infiltrating breast cancer and the surrounding normal tissue revealed a different clonal composition of the tumor and adjacent tissue, with TILs showing higher T-cell clonality as compared with T cells infiltrating normal tissue [66]. The clonal configuration was more similar between circulating T cells and normal breast tissue than tumor-infiltrating T cells. However, both the tumor and normal breast tissue displayed the presence of lymphocytes with enriched sequences, absent or poorly expressed in other tissues, implying the existence of a compartmentalization of T cells [66].

Overall, most studies report that high TCR diversity is often associated to a better disease progression, whereas low diversity correlates with more aggressive phenotypes [67]. However, what emerges is the urging need for a deep understanding of the complex crosstalk between TAAs and the host adaptive immunity, critical for finding therapeutic targets and developing effective immunotherapies. Considering all the emerging findings, very diverse scenarios can be expected with TILs either having a homogeneous TCR repertoire throughout distinctive tumor areas, or showing heterogeneity originating from longitudinal changes in the rate and nature of neo-Ags a within the same tumor.

Another feature to be taken into consideration is the frequency of public and private TCR sequences. In particular, the latter have been most often associated with a more specific antitumor response, with respect to public sequences, many of which are present also in non-tumor tissues and may likely reflect a non-specific response [68]. Li et al. suggested that the population of tumor-infiltrating T cells retain a large fraction of public clonotypes which show shorter CDR3 regions compared to private T cells, and are less likely to bind neo-epitopes according to the hydrophobicity analyses [69]. Interestingly, the middle 3 amino acids of the private CDR3 motifs contain significantly higher portion of hydrophobic residues than those of the public motifs, and hydrophobicity has been described as a hallmark of immunogenic neo-epitopes, suggesting that private CDR3 sequences might have higher potential for tumor-Ag recognition [69, 70]. Finally, as emerging from recent findings by Zhuo et al., healthy elder individuals similarly to cancer patients, display a very poor TCR diversity within the periphery, revealing alteration of TCR repertoire in aging and oncogenesis, suggesting a possible use of circulating TCR profiling as marker of immune cell fitness in clinical practice [71].

### Role of the TCR repertoire in response to cancer vaccination

In the framework of Ag specific cancer vaccines, we have reported that peptide-vaccination combined with chemotherapy is able to induce a diversification of Melan-A-specific T-cell repertoire in the peripheral blood of long-surviving melanoma patients, with a turn-over of new polyfunctional and tumor-reactive clonotypes [72, 73]. The increased diversity of TCR repertoire was not observed in patients treated with the peptide vaccination alone [72]. Noteworthy, diversity may be related to the nature of the tumor Ags, since the TCR repertoire specific for gp100, the other peptide used in the vaccine formulation, was oligoclonal irrespective of the combined dacarbazine treatment [74, 75]. In agreement, Stuge and co-workers observed a diversified Melan-A-specific CD8^+^ repertoire expanded after peptide-vaccination, although a significant proportion of these cells was unable to lyse tumor cells [76]. TCR repertoire diversity may also be affected by the vaccine formulation as reported by Connerotte et al. who described that only the vaccine formulation including MAGE-3-peptide-pulsed dendritic cells was able to generate a polyclonal T-cell response in melanoma patients with tumor regression, whereas MAGE-3 peptide or ALVAC mini-MAGE1/3 vaccinations induced a monoclonal T-cell response [77]. Conversely, a different study correlated the diversification of TCR repertoire to the use of the natural, rather than the modified, Melan-A 26–35 peptide in the vaccine formulation [78].

More recently [79], TCRαβ sequencing and immunophenotyping of circulating T cells form patients with metastatic melanoma receiving a personalized neo-Ag cancer vaccine, combined with anti-PD-1 therapy, showed that prolonged progression-free survival (PFS) was strongly associated with increased TCR clonality which did not change over treatment, a behavior referred to as repertoire stability [79, 80]. Although the frequency of persistent clones correlated to the frequency of effector-memory CD8^+^ and CD4^+^ T cells, this capability was shared by both neoAg-specific and vaccine-expanded clones.

An increased diversity of the circulating TCR repertoire, correlating with longer survival, was observed in either lung [81] and colorectal [82] cancer patients treated with cancer peptide vaccines. Interestingly, poor responders did not show any augmented repertoire diversity. An important question is whether the peripheral TCR repertoire mirrors the TCR changes occurring at the tumor site. Tamura and co-workers reported that advanced CRC patients showing reduced TCR diversity at the tumor site exhibited longer PFS after vaccine and chemotherapy treatment [82]. Moreover, TCR clones directed against tumor Ags were untraceable in the periphery before treatment, but they became detectable after vaccination. This suggests that the expanded T-cell populations could have been activated in the lymph nodes near the vaccine injection site and allowed to circulate in the blood, thus reflecting accumulation and activation of certain T-cell populations at the tumor site adequately fit to overcome the inhibitory signals provided by the TME. In a recent study, patients with metastatic deficient mismatch repair (dMMR) tumors, treated with anti-PD-1 and vaccinated with an adenoviral platform (Nous-209) encoding shared neo-Ags, showed expansion and diversification of the TCR repertoire as well as an increase in tumor-infiltrating T cells with an effector memory signature [83]. These findings suggest a way to overcome resistance to anti-PD-1 treatment by enhancing TIL immunogenicity and diversity, induced by the vaccine.

Thanks to the advent of NGS approaches, whole cancer exomes can be sequenced and compared with healthy tissue (germline exome), providing new perspectives to convert the patient individual alterations into a personalized vaccine [84, 85]. Such an approach may overcome the tremendous cancer heterogeneity and increase the probability of generating an effective tumor-specific immune response.

### TCR repertoire diversity in response to ICIs

The employment of ICIs has been proven to increase survival of patients with different types of cancer [86–91]. Nevertheless, the limited proportion of patients who benefit from ICIs highlights the requirement to identify patient-specific immunological features accounting for efficient treatment response. By mirroring the specificity and strength of human immune response, the TCR repertoire represents a “dynamic fingerprint” of the events evolving at the tumor site. Hence, TCR analysis can provide valuable insights to improve ICI efficacy and safety, guiding patient stratification and allowing immune-monitoring during therapies. So far, biomarkers of ICI success have been focused mainly on the intratumoral expression of inhibitory ligands, including PD-L1, on the tumor mutational burden and on the presence of infiltrating T cells. More recently, several studies have tried to explore the correlation between the modification of both TCR diversity and clonality with the establishment of a protective tumor-specific response during ICI therapy, leveraging the possibility to trace tumor-specific T cells by TCR profiling. Most of these investigations employed circulating T cells, due to the difficulty of obtaining longitudinal tumor samples, nevertheless TCR profiling of infiltrating T cells is also emerging, highlighting the different impact on TCR diversity exerted by the mAbs targeting the two major ICIs, namely CTLA-4 and PD-1 [92].

In this paragraph we aim at summarizing recent evidence supporting precise TCR repertoire profiling as a novel predictive biomarker of ICI response to optimize patient benefit, minimize toxicity, and guide combination approaches. Specifically, we describe the role of the endogenous TCR profiling before therapy and after the two major immune checkpoint treatments, mainly in melanoma patients.

Initial investigations on the effect of baseline TCR repertoire on melanoma patients treated with anti-CTLA-4 or anti-PD-1, showed that a high diversity in peripheral blood, was associated with good clinical outcomes. Specifically, patients with clinical benefit after CTLA-4 blockade exhibited higher richness and evenness in their baseline TCR repertoires compared to non-responders, suggesting that a more diverse TCR repertoire may be critical to enhancing clinical response to anti-CTLA4 treatment [13].

According to a recent retrospective study [93], melanoma patients responding to anti-PD-1 or anti-CTLA-4 therapy exhibited low TCR diversity, in pre-treatment peripheral T cells. In particular, the authors showed that low diversity evenness was differentially predictive of poor outcome in patients treated with anti-CTLA-4 and longer PFS in those treated with anti-PD-1 [93]. This study confirmed previously observed data from melanoma biopsies of patients treated with anti-CTLA-4 followed by PD-1 blockade, namely that a more clonal baseline TCR repertoire was predictive of response to PD-1, but not to CTLA-4 blockade [94]. Moreover, Riaz and colleagues have reported a reduced evenness without a significant change in terms of richness, which was amplified by nivolumab irrespective of the beneficial effect, in patients receiving immunotherapy [15]. The authors performed a longitudinal multi-omics analysis on a large cohort of melanoma patients, either naïve or previously treated with anti-CTLA-4, revealing how tumor co-evolves with the antitumoral immune response during anti-PD-1 treatment. They found a positive association of mutational load and treatment response in patients naïve to prior CTLA-4 administration. Temporal changes in intratumoral TCR repertoire revealed a decreased evenness without a significant change in terms of richness in responder patients not previously treated with ipilimumab. This is consistent with the expansion and accumulation of specific T-cell clonotypes in response to tumor-antigen recognition [15].

Another critical question is how the broadening or expansion of the TCR repertoire can be considered a biomarker of patient response to treatment with ICIs. In a retrospective study on melanoma and prostate cancer patients, CTLA-4 blockade has been described to support a vigorous renovation of the TCR repertoire by inducing the occurrence of an overall enlarged repertoire diversity. Cha et al., in 2014 described how patients with metastatic castration-resistant prostate cancer and metastatic melanoma treated with anti-CTLA-4 showed both a circulating repertoire enlargement and a loss of T-cell clonotypes, with an overall renewal of the TCR repertoire which evolved during the treatment, along with increased TCR diversity [19]. However, the increase in the number of clonotypes was not related to the clinical outcome, although an enhanced OS was correlated to the preservation of clones showing high frequency before therapy. Differently, the occurrence of clonotypes with the highest frequency dropped following the treatment in patients with short OS. These observations indicate that CTLA-4 blockade provokes a broadening of the TCR repertoire and that a lower incidence of clonotypes lost after therapy, is associated with better clinical outcomes. In particular, this is indicative of preservation of high-frequency clonotypes along the blockade therapy, which may provide a baseline high-avidity antitumor response [19]. Robert et al., analyzed the TCR features in the peripheral blood-derived T cells from patients treated with tremelimumab, an anti-CTLA-4 mAb, before and after therapy, observing a general increase in unique productive TCR V-beta CDR3 sequences in almost all patients [95].

Differently from what observed with anti-CTLA-4 therapies, blockade of PD-1 has been described to expand specific clones, resulting in a less diverse T-cell populations in metastatic melanoma patients [96]. In particular, Tumeh and colleagues [97] showed higher TCR clonality and reduced frequency of the less diverse population in responding patients as compared with those with disease progression. Also, tumor tissue-resident memory T cells displayed a ten-times enrichment of specific clones after anti-PD-1 therapy in responding patients compared with progressors, which implies a tumor-specific response to therapy for these patients. Remarkably, baseline TCR clonality was not particularly linked to the density of TILs, which suggests that also patients with poor T-cell tumor-infiltration might still benefit from anti-PD-1 therapy, if these cells have a restricted TCR clonality. The analysis of TCR repertoires before and during anti-PD-1 therapy indicated that in responding patients, proliferation of intratumoral CD8^+^ T cells is directly linked to a decrease of the tumor dimension. Of note, the baseline clonal TCR repertoire displayed by T cells of patients responsive to PD-1 blockade required the pre-existence of CD8^+^ T cells emerging from the area surrounding the tumor and expressing juxtaposed PD-1 and PD-L1. Enhanced T-cell clonality in anti-PD-1 responding patients was also reported in NSCLC, glioblastoma, metastatic bladder cancer and pancreatic cancer [36, 98, 99]. Two recent studies by Valpione et al. [100] and Fairfax et al. [101] on metastatic melanoma patients receiving ICI treatments, revealed an early (3 weeks) increase of circulating TCR repertoire clonality in patients showing durable clinical benefit. Moreover, the authors of the two independent studies observed the specific expansion of a distinctive subset of cytotoxic memory effector peripheral T cells (T_IE_), known to infiltrate tumors. These findings encourage non-invasive and early monitoring of patients receiving ICIs to anticipate clinical response to the treatment. The importance to frame early time points and quantify the dynamics of TCR repertoire during immunotherapy, was also confirmed in a syngeneic mouse model of CRC, where the authors identified a transient and initial increase in clonality in parallel with a decreased diversity which gradually receded [102]. More recently, Wucherpfennig group published an elegant study where, by designing a powerful experimental set up, the authors temporally characterized the response of T-cell subsets to anti-PD-1 and anti-CTLA-4 treatments, both in the tumor and blood of head and neck cancer patients, in a neoadjuvant setting [103]. ScRNA- and TCR-seq of T cells, pre- and post-treatment, allowed the direct identification of a subset of tumor-infiltrating CD8^+^ T cells, clonally expanded during immunotherapy, with elevated tumor tissue-resident memory and cytotoxicity programs, deemed as the predominant T-cell population responding to neoadjuvant ICIs.

Whether T-cell response to ICI relies on pre-existing tumor infiltrating clonotypes or, on a distinct reservoir of T-cell clones recruited to the tumor site, is not yet clearly defined. By employing cutting-edge technologies, including paired scRNA- and TCR-seq, Yost and colleagues found that the expansion of T cells upon checkpoint blockade stems from distinct T-cell clones that may have just infiltrated the tumor bed [18]. These data highlight how high-resolution TCR profiling has become an invaluable tool to gain insights into fundamental biological processes, by allowing dynamical T-cell tracing in time and space.

Immune checkpoint blockade is correlated to a series of immune-related adverse events (IRAE), whose underlying mechanisms have not been defined yet. In the above-mentioned investigation [18] a substantial divergence was observed in the total unique productive TCR V-beta CDR3 sequences between patients undergoing toxicity, as compared with patients without significant adverse effects. Therefore, the CTLA-4 blockade-associated expansion of the extent of single TCR sequences in the peripheral blood was linked to the generation of autoimmunity and inflammation. Moreover, an ipilimumab-associated diversification in the TCR repertoire has been mostly observed in patients with IRAE compared to patients without adverse effects. In particular, an initial broadening in the repertoire has been shown to happen within 2 weeks of treatment, preceding the IRAE onset. Also, PFS response to ipilimumab has been associated with increased TCR diversity, showing how a prompt diversification in the immune repertoire immediately after checkpoint blockade can be both detrimental and beneficial for cancer patients [104].

Knowledge about multiple clinical variables is essential for precision immune-oncology and clinical decision making. By examining the clinical characteristics of patients with melanoma treated with first-line anti-PD-1 mAb, Zena et al. observed age-related effects on TCR repertoire evolution and T_IE_ cell expansion, showing that age influences T-cell reinvigoration by ICI therapy and, therefore, that it should be included among the biomarkers used to monitor responses to immunotherapy [105]. Additional research suggested that T-cell clones shared between blood and tumor (overlapping clones) are those more informative on the clinical outcome, as a higher frequency of overlapping clones within peripheral CD8^+^ T cells before anti-PD-1 treatment was associated with a favorable clinical response [106–108].

### Immunological qualities of cancer chemotherapy, when chemotherapy helps immunotherapy

The combination of ICI with chemotherapy, referred as chemoimmunotherapy, has changed the standard of care in clinical practice and the neoadjuvant setting has recently achieved unprecedented clinical success, rising increasing interest for the identification of the underlying molecular mechanisms. Clinical indications from ongoing trials in locally advanced stages of NSCLC, has revealed promising results pointing to a complete pathologic response (CPR) in 63% of patients treated with neoadjuvant chemoimmunotherapy [109, 110]. As largely discussed in the above paragraph, TCR profiling is becoming a reliable tool to survey antitumor response and a promising biomarker for immunotherapy [79], also in NSCLC [49]. A seminal explorative investigation from Casarrubios and colleagues defined the TCR repertoire dynamics, with a temporal and spatial resolution, in NSCLC patients receiving neoadjuvant chemoimmunotherapy [111]. Precise TCR-seq of cancer-infiltrating T cells showed that the presence of top 1% clonal space and TCR evenness imply a high tumor immunogenicity and are associated with CPR in patients receiving neoadjuvant chemoimmunotherapy. Furthermore, the same tissue top 1% clones were expanded also in peripheral blood, suggesting that systemic immunosurveillance could explain the observed complete clinical response and protection from relapse [111].

By coupling TCR profiling with RNA-seq on infiltrating immune cells from 12 surgical resected NSCLC patients, Hui et al. investigated the tumor immune transcriptomic profiles and their association with the clinical response to neoadjuvant pembrolizumab and chemotherapy at single-cell resolution [112]. This led to the identification of several key events associated to a positive clinical outcome, including tertiary lymphoid structure development and expansion of intratumoral CD4^+^ T and peripheral CD8^+^ T-cell clones [112]. In particular, by following the migration trajectories of expanded tumor tissue-resident memory T-cell clones, they suggested that the reinvigoration of TAA clonotypes occurs within the circulating compartment which dynamically exchange with the tumor lesion, further supporting the monitoring of peripheral TCR repertoire diversity and clonality as non-invasive predictors of patient response and survival in NSCLC [113].

Chemoimmunotherapy is a recognized treatment for triple-negative breast cancer (TNBC) [114, 115], however not all chemoimmunotherapy regimens have improved the clinical outcomes for patients with metastatic disease [116, 117] suggesting that different ICI/chemotherapeutic combination can distinctively shape antitumor response. Chun et al., have recently provided the first comparison of the effects of anti-PD-1 treatment combined with paclitaxel or capecitabine on T-cell subset repertoire during first or second-line treatment of patients with metastatic TNBC [118]. No differences in TCR repertoire clonality were detected after the administration of the different combinations within the circulating and tissue compartment, however, using a novel computational approach, the authors found that paclitaxel plus anti-PD-1 induces the generation of a higher number of novel clonotypes than capecitabine. Although additional evaluations are needed to understand the mechanism elicited by the different chemotherapy backbones on immune cell populations, emerging data offer valuable insights for future characterization of specific chemoimmunotherapy combinations.

One hypothesis that may explain the success of this approach is that the modifications induced by chemotherapy within the TME can support a specific T-cell mediated antitumor response, further improved by immune-stimulating agents [119]. This has been the case for a broadly used class of chemotherapeutics, specifically, alkylating agents. Alkylating chemotherapy includes a class of DNA-damaging compounds that covalently modify DNA by either methylating distinctive bases or generating inter-strand or intra-strand alkyl crosslinks. In a murine genetically engineered model of BRCA1 breast cancer, the combination of anti-CTLA-4 and anti-PD-1 therapies with cisplatin-based chemotherapy has resulted in improved survival [120]. DNA damaging agents able to induce double-strand breaks could probably synergize with PD-1 blockade by facilitating innate immune recognition. Studies involving several chemotherapeutics and more targeted DNA damaging agents are under way and can contribute to elucidate whether this mechanism plays a critical role in human tumors. In the case of triazenes, immunogenic mutations are produced by methylation of O6-guanine of DNA and are confidently associated with a broadening of the antigenic breadth and clonal diversity of antitumor immunity [121]. Therefore, it can be postulated that mutation-dependent neo-Ags, obtained by appropriate pharmacological intervention, may represent a novel approach for enhancing the therapeutic efficacy of selected ICIs in cancer patients. Recently, from a phase II study evaluating the use of pembrolizumab in metastatic CRC patients with chemo-refractory mismatch repair–proficient (MMRp) and O6-methyl-guanine-DNA-methyltransferase promoter methylated metastatic CRC, emerged that temozolomide may modulate the immunogenicity and enhance pembrolizumab efficacy [122]. Altogether, this evidence highlight the relevance of investigating chemotherapy associated TCR repertoire in tumors to better establish the chemo-immunotherapeutic combinations.

### New technical advancements for the TCR repertoire analysis

The above-described major advancements in T-cell autologous or immunotherapy-induced response to cancer, has been made possible thanks to the advent of high-throughput technology, able to study not only mutational, gene expression and epigenetic landscapes, but also the immune cell landscape and TCR repertoire.

TCR sequencing allows indeed to characterize the entire V(D)J sequences of T cells retrieved from one or multiple T-cell populations from a single or multiple samples, namely the entire TCR repertoire. Very recent advancements have brought the development of single-cell technologies, also applied to the characterization of TCR, that may be paired with transcriptomic profiles or chromatin accessibility assays, in the so-called multi-modal experiments. In the next paragraphs we will provide an overview of the latest technical and computational improvements in T-cell sequencing, highlighting both challenges and strengths of each approach.

### Bulk sequencing

At the beginning of NGS revolution, bulk TCR-seq has been widely used to investigate and characterize the TCR repertoire in terms of diversity, clonality and antigen specificity [9]. Many commercial kits have been made available from multiple companies to retrieve TCR sequences from dissociated tissues or flow-sorted cell populations, using both genomic DNA (gDNA) and messenger RNA (mRNA) as starting material. Both gDNA and mRNA have their own pros and cons: the former was recommended for the stable number of TCR copies (one TCRβ rearrangement per cell) that enables direct quantification, but it does not consider the allelic exclusion, thus reducing sensitivity and clonotypes diversity evaluation. On the other hand, the mRNA-based approach, although less stable, is not affected by the allelic exclusion issue and has increased sensitivity, also thanks to the possibility to employ Unique Molecular Identifiers (UMI) to minimize amplification biases and sequencing errors. Besides, mRNA expression levels can differ between cells (multiple copies of TCR per cell), sometimes leading to difficulties in the quantification of clonal expansion [123]. The major downside of bulk TCR-seq is that it can only provide information about a single TCR chain, thus failing in characterizing the heterodimeric receptor subunits pairing, a fundamental determinant of TCR Ag specificity. This is due to the technical steps required for TCR sequences retrieval and amplification. Both multiplex PCR and 5’ Rapid Amplification of cDNA Ends (RACE) methods introduce some biases hindering the αβ pairing in the downstream analyses, thus preventing researchers to fully appreciate the potential of TCR repertoire portraying in improving the quality of functional and clinical studies, especially when considering rare T-cell populations [124].

### Single-cell sequencing

Recently, NGS-based single-cell TCR-seq has emerged, revealing that differently from bulk sequencing, single-cell methodologies can retrieve cells individually from a pool and generate a uniquely barcoded sequencing library, thanks to microfluidics or nanowell-based technologies [125, 126]. Moreover, UMIs are added to the pre-amplification step, to ensure the correct quantification of mRNA transcripts and TCR from single cells that can be captured and sequenced simultaneously, allowing the identification and functionality of subpopulations, thanks to the sequence of TCRαβ pairs, transcriptional trajectories and states of T-cell subsets [127]. Although scTCR-seq brings the opportunity of having full length sequences per cell, it is not free from sequencing errors. To date, methods for capturing and amplifying the full TCR sequence in single cells, relies on methods used in bulk sequencing such as the above-described PCR-based methodology, thus sharing the same pitfalls [16].

### Spatial transcriptomics and TCR

Alongside single-cell sequencing, in the last years the power of spatial transcriptomics has been growing rapidly, and being compatible with archival tissues, has opened new avenues for retrospective analyses of samples available in pathology units and bio-banks. Exploration of spatial transcriptomic findings will help model tissue organization and identify the mechanisms involved in tissue homeostasis and its deregulation as occurs in cancer. The possibility to study gene expression on intact tissue, by maintaining spatiality, can provide meaningful information not only about cell states and phenotype, but also on the crosstalk between cellular and non-cellular components, ultimately determining signaling to which cells are exposed [128]. Moreover, imaging tools can be leveraged to identify cells or tissue areas expressing specific surface markers that, coupled with gene expression, can guide downstream analyses [129]. Sudmeier and colleagues successfully paired spatial transcriptomics with TCR-seq by adapting the 10X Visium experimental protocol, revealing how tumor infiltrating CD8^+^ T-cell subpopulations have different phenotypes and localize in specific niches within brain metastasis tumor core or parenchyma, fostering the development of more specific immunotherapeutic strategies [42, 130]. Recently, a novel methodology, referred as to Slide-TCR-seq, has been developed by Liu et al. for sequencing whole transcriptomes and TCRs within intact tissue [42, 43, 130]. The unprecedented opportunity to explore the TCR repertoire dynamics in their spatial context will be instrumental in understanding how the anatomical distribution of T cells and their partners shapes immune response, particularly within the dysregulated TME.

### Computational analysis of TCR repertoires

Since TCR-seq has been widely adopted, many computational tools for primary and secondary data analysis have emerged. Among primary data processing tools, most popular are MiXCR, IMSEQ, RTCR, IMGT/HighV-QUEST, IgBlast, MiGEC, ImmunoSEQ, SODA2 and iHMMune-align [123, 131–138]. Each of them uses a different strategy to identify V(D)J segments and annotate the CDR3 sequence: some use the BLAST algorithm, others a Hidden Markov Models-based approach or an ad-hoc built algorithm to exactly match the V(D)J sequences from databases of known TCR sequences and extract the CDR3 region nucleotides. Moreover, in recent years, the Immcantation computational framework has been developed. This framework relies on ad-hoc built and third-party pieces of software, providing a complete computational environment to carry out both data processing and secondary data analysis [139].

One of the most used computational tools is MiXCR, that allows to fine tune processing parameters to adjust the pipeline to data types and desired outputs. Furthermore, it can also reconstruct TCR sequences starting from RNA-seq or WES experiments that can yield another piece of information from the same data, although with major limitations compared to proper TCR-seq experiments [131]. Other strategies to reconstruct TCR sequences from scRNA-seq data have been also developed over time. These strategies rely on Bayesian statistics or proper mapping of reads against a database of all known TRAV and TRBV sequences [140–142].

To gain helpful information, scientists usually analyze the TCR repertoire in terms of diversity and clonality of T-cell clonotypes, along with the different usage of V/J genes and spectral information. Among the measures of clonotypes diversity, Shannon, Simpson and Gini diversity indices have been adopted and are often considered together to get full insight from data, as they weight species richness and evenness differently [12]. V/J genes usage is often taken into consideration because of the tendency of being biased toward specific rearrangements in particular settings such as cancer and autoimmunity [143, 144]. Most employed software package for the described analyses are VDJTools and ImmunArch [145].

Profiling the TCR repertoire at precise molecular and spatial resolution, either at bulk and single-cell level, is opening a new era in the identification of T-cell dynamics, differentiation and response trajectories, with major implications in health and disease, as thoroughly discussed in this review. Technical improvements and high cost, particularly for single-cell methods, still represent challenges to address, however new opportunities are also emerging, in particular the combination of the discussed methodologies with orthogonal methods, that may lead to an even finer understanding of T-cell response and instigate new therapeutic approaches.

## Conclusions

**Fig. 1:**
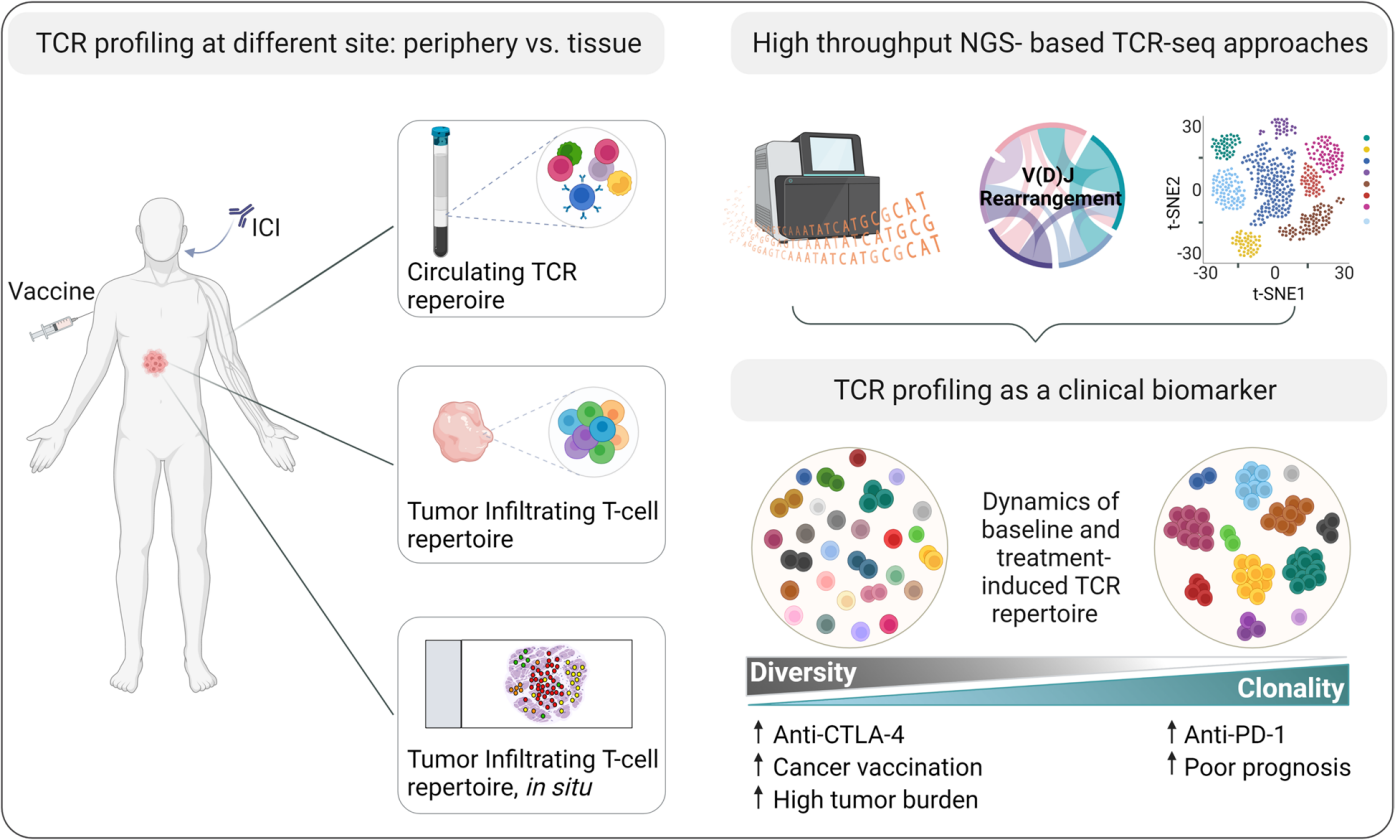
Advancement in TCR repertoire profiling and clinical applications. T cells from different body compartments, including blood or tissues can be subjected to accurate TCR profiling. High-throughput sc-TCR sequencing combined with spatial and transcriptomic information can offer a plethora of key insights to decipher the complex nature of antitumor immune responses and track T cell dynamics over time and space. TCR repertoire meters, including diversity and clonality can be derived and used as biomarkers of prognosis and response to immunotherapy. Created with BioRender.com

TCR profiling is a powerful novel approach in the analysis of host–tumor interaction and response to therapies (Fig. 1). However, what emerges from the reported observations is a complex picture where the antitumor immune response is continually shaped by several factors, resulting from the interplay of tumor genomic characteristics, local microenvironment modulation, and intrinsic antitumor T-cell capabilities. The development of multi-modal experiments, integrating TCR repertoire sequencing at single-cell resolution will be of central importance for precise T-cell phenotyping and functionality, identification of transcriptional T-cell trajectories and specific tracking of distinct T-cell subsets in periphery and at tissue level. Several evidence show alterations of TCR repertoire in cancer and response to therapy, encouraging the clinical use of repertoire measurements for progression monitoring, assessment of response to treatment and patient stratification. As far, only few reliable biomarkers are accessible for clinicians to improve patient selection and treatment efficacy for immunotherapy. Thus, validation of TCR repertoire analysis combined with the latest advances in machine-learning approaches, that will potentially uncover the tumor Ag specificity of the immune repertoire, will be fundamental to improve cancer care and ultimately for the development of novel strategies to improve patient outcomes.

## Data Availability

Not applicable.

## Abbreviations

Ag: Antigen
BrM: Brain Metastasis
CAR-T: Chimeric Antigen Receptor-T
CRC: ColoRectal Cancer
CPR: Complete Pathologic Response
HLA: Human Leukocyte Antigen
ICI: Immuno Checkpoint Inhibitor
MHC: Major Histocompatibility Complex
NGS: Next-Generation Sequencing
NSCLC: Non-Small Cell Lung Cancer
OS: Overall Survival
PFS: Progression-free survival
RACE: Rapid Amplification of cDNA Ends
RNA-seq: RNA-sequencing
Sc: Single cell
TAA: Tumor Associated Antigen
TCR-seq: TCR-sequencing
TCR: T-Cell Receptor
TIL: Tumor Infiltrating Lymphocyte
TME: Tumor MicroEnvironment
TNBC: Triple-Negative Breast Cancer
UMI: Unique Molecular Identifier

## Glossary

Clonality: It is an estimate of clonal expansion, and is defined as the probability that two independently identified sequences originate from the same clone. It is based on the normalized Shannon entropy [146], and it is inversely related to the diversity of T-cell clones.
DE_50_: The diversity evenness score represents an index of clonality [147]. It describes, when the number of reads is summed up in rank order (from high to low), how many unique reads exist within 50% of in-frame reads. The lower the number is, the higher the clonality.
Diversity: Repertoire diversity takes into account the clonal composition, corresponding to the number of unique TCR sequences (richness) and the distribution of these sequences (evenness). Diversity can be mathematically derived from information theory used in ecology to measure ecosystem biodiversity and quantified by several indices, as reviewed by Chiffelle et al (12).
Evenness: Distribution of the frequencies of the unique TCR sequence (richness) of a repertoire. It allows to discriminate among repertoires with different clonal compositions, i.e., those that contain very few expanded clones with several rare ones, and those which are evenly distributed [148].
Gini coefficient: It is a common index used to derive the clonal distribution of a repertoire [149]. It quantifies inequality, i.e., evenness rather than the richness.
Inverse Simpson index: This index is used when dataset contains high-frequency reads [150]. High values suggest an even distribution of TCR clones, whereas low values indicate enrichment of T-cell clones.
Jaccard index: It is a measure of similarity/dissimilarity between two repertoires [151]. It is defined as the size of overlapping species divided by the size of both compared repertoires. It varies from 0 (no overlap) to 1 (perfect overlap).
The Morisita–Horn index: It is a measure of similarity (overlap) between two repertoires [152]. It takes into account both the number and abundance of shared TCRs between the two repertoires. It varies from 0 (no overlap) to 1 (perfect overlap).
Pielou’s index: It measures the clonality of a repertoire, it is defined by the ratio between the Shannon entropy and the maximization of the diversity distribution of species within a sample [153].
Richness: Number of unique TCR sequences, generated by the random and error-prone rearrangements of the V and J segments of the TCRα and V, D, and J segments of the TCRβ genes in the thymus.
Shannon entropy index: A common measure of TCR diversity which takes into account sample richness and evenness [154]. It ranges between 0 and 1, where 1 indicates the most diversity, and 0 absence of diversity. When a repertoire is composed of evenly distributed clones, the Shannon entropy reaches his maximum.

## References

[1] Dong, Zheng L, Lin J, Zhang B, Zhu Y, Li N (2019). Structural basis of assembly of the human T cell receptor-CD3 complex. Nature.

[2] Davis MM, Krogsgaard M, Huse M, Huppa J, Lillemeier BF, Li QJ (2007). T cells as a self-referential, sensory organ. Annu Rev Immunol.

[3] Lanz AL, Masi G, Porciello N, Cohnen A, Cipria D, Prakaash D (2021). Allosteric activation of T cell antigen receptor signaling by quaternary structure relaxation. Cell Rep.

[4] Stritesky GL, Jameson SC, Hogquist KA (2012). Selection of Self-Reactive T Cells in the Thymus. Annu Rev Immunol.

[5] Alcover A, Alarcón B, Di Bartolo V (2018). Cell Biology of T Cell Receptor Expression and Regulation. Annu Rev Immunol.

[6] Max EE, Seidman JG, Leder P (1979). Sequences of five potential recombination sites encoded close to an immunoglobulin kappa constant region gene. Proc Natl Acad Sci U S A.

[7] Lythe G, Callard RE, Hoare RL, Molina-París C (2016). How many TCR clonotypes does a body maintain?. J Theor Biol.

[8] Arstila TP, Casrouge A, Baron V, Even J, Kanellopoulos J, Kourilsky P (1999). A direct estimate of the human alphabeta T cell receptor diversity. Science.

[9] Qi Q, Liu Y, Cheng Y, Glanville J, Zhang D, Lee JY (2014). Diversity and clonal selection in the human T-cell repertoire. Proc Natl Acad Sci U S A.

[10] Tanno H, Gould TM, McDaniel JR, Cao W, Tanno Y, Durrett RE (2020). Determinants governing T cell receptor α/β-chain pairing in repertoire formation of identical twins. Proc Natl Acad Sci U S A.

[11] Sun X, Nguyen T, Achour A, Ko A, Cifello J, Ling C (2022). Longitudinal analysis reveals age-related changes in the T cell receptor repertoire of human T cell subsets. J Clin Invest.

[12] Chiffelle J, Genolet R, Perez MA, Coukos G, Zoete V, Harari A (2020). T-cell repertoire analysis and metrics of diversity and clonality. Curr Opin Biotechnol.

[13] Postow MA, Manuel M, Wong P, Yuan J, Dong Z, Liu C (2015). Peripheral T cell receptor diversity is associated with clinical outcomes following ipilimumab treatment in metastatic melanoma. J Immunother Cancer.

[14] Bradley P, Thomas PG (2019). Using T Cell Receptor Repertoires to Understand the Principles of Adaptive Immune Recognition. Annu Rev Immunol.

[15] Riaz N, Havel JJ, Makarov V, Desrichard A, Urba WJ, Sims JS (2017). Tumor and Microenvironment Evolution during Immunotherapy with Nivolumab. Cell.

[16] Han A, Glanville J, Hansmann L, Davis MM (2014). Linking T-cell receptor sequence to functional phenotype at the single-cell level. Nat Biotechnol.

[17] Azizi E, Carr AJ, Plitas G, Cornish AE, Konopacki C, Prabhakaran S (2018). Single-Cell Map of Diverse Immune Phenotypes in the Breast Tumor Microenvironment. Cell.

[18] Yost KE, Satpathy AT, Wells DK, Qi Y, Wang C, Kageyama R (2019). Clonal replacement of tumor-specific T cells following PD-1 blockade. Nat Med.

[19] Cha E, Klinger M, Hou Y, Cummings C, Ribas A, Faham M (2014). Improved survival with T cell clonotype stability after anti-CTLA-4 treatment in cancer patients. Sci Transl Med.

[20] Holt RA, Jones SJ (2008). The new paradigm of flow cell sequencing. Genome Res.

[21] Shendure J, Ji H (2008). Next-generation DNA sequencing. Nat Biotechnol.

[22] Woodsworth DJ, Castellarin M, Holt RA (2013). Sequence analysis of T-cell repertoires in health and disease. Genome Med.

[23] Linnemann C, Heemskerk B, Kvistborg P, Kluin RJ, Bolotin DA, Chen X (2013). High-throughput identification of antigen-specific TCRs by TCR gene capture. Nat Med.

[24] Robins HS, Srivastava SK, Campregher PV, Turtle CJ, Andriesen J, Riddell SR (2010). Overlap and effective size of the human CD8+ T cell receptor repertoire. Sci Transl Med.

[25] Fozza C, Barraqueddu F, Corda G, Contini S, Virdis P, Dore F (2017). Study of the T-cell receptor repertoire by CDR3 spectratyping. J Immunol Methods.

[26] Liaskou E, Klemsdal Henriksen EK, Holm K, Kaveh F, Hamm D, Fear J (2016). High-throughput T-cell receptor sequencing across chronic liver diseases reveals distinct disease-associated repertoires. Hepatology.

[27] Waldman AD, Fritz JM, Lenardo MJ (2020). A guide to cancer immunotherapy: from T cell basic science to clinical practice. Nat Rev Immunol.

[28] Morgan RA, Dudley ME, Wunderlich JR, Hughes MS, Yang JC, Sherry RM (2006). Cancer regression in patients after transfer of genetically engineered lymphocytes. Science.

[29] Harris DT, Hager MV, Smith SN, Cai Q, Stone JD, Kruger P (2018). Comparison of T Cell Activities Mediated by Human TCRs and CARs That Use the Same Recognition Domains. J Immunol.

[30] De Mattos-Arruda L, Vazquez M, Finotello F, Lepore R, Porta E, Hundal J (2020). Neoantigen prediction and computational perspectives towards clinical benefit: recommendations from the ESMO Precision Medicine Working Group. Ann Oncol.

[31] Chandran SS, Klebanoff CA (2019). T cell receptor-based cancer immunotherapy: Emerging efficacy and pathways of resistance. Immunol Rev.

[32] McLane LM, Abdel-Hakeem MS, Wherry EJ (2019). CD8 T Cell Exhaustion During Chronic Viral Infection and Cancer. Annu Rev Immunol.

[33] Scheper W, Kelderman S, Fanchi LF, Linnemann C, Bendle G, de Rooij MAJ (2019). Low and variable tumor reactivity of the intratumoral TCR repertoire in human cancers. Nat Med.

[34] Simoni Y, Becht E, Fehlings M, Loh CY, Koo SL, Teng KWW (2018). Bystander CD8. Nature.

[35] Meier SL, Satpathy AT, Wells DK (2022). Bystander T cells in cancer immunology and therapy. Nat Cancer.

[36] Gerlinger M, Rowan AJ, Horswell S, Math M, Larkin J, Endesfelder D (2012). Intratumor heterogeneity and branched evolution revealed by multiregion sequencing. N Engl J Med.

[37] Woolaver RA, Wang X, Krinsky AL, Waschke BC, Chen SMY, Popolizio V (2021). Differences in TCR repertoire and T cell activation underlie the divergent outcomes of antitumor immune responses in tumor-eradicating versus tumor-progressing hosts. J Immunother Cancer.

[38] Idos GE, Kwok J, Bonthala N, Kysh L, Gruber SB, Qu C (2020). The prognostic implications of tumor infiltrating lymphocytes in colorectal cancer: a systematic review and meta-analysis. Sci Rep.

[39] Lee N, Zakka LR, Mihm MC, Schatton T (2016). Tumour-infiltrating lymphocytes in melanoma prognosis and cancer immunotherapy. Pathology.

[40] El Bairi K, Haynes HR, Blackley E, Fineberg S, Shear J, Turner S (2021). The tale of TILs in breast cancer: A report from The International Immuno-Oncology Biomarker Working Group. NPJ Breast Cancer.

[41] Li F, Li C, Cai X, Xie Z, Zhou L, Cheng B (2021). The association between CD8+ tumor-infiltrating lymphocytes and the clinical outcome of cancer immunotherapy: A systematic review and meta-analysis. EClinicalMedicine.

[42] Sudmeier LJ, Hoang KB, Nduom EK, Wieland A, Neill SG, Schniederjan MJ (2022). Distinct phenotypic states and spatial distribution of CD8. Cell Rep Med.

[43] Liu S, Iorgulescu JB, Li S, Borji M, Barrera-Lopez IA, Shanmugam V (2022). Spatial maps of T cell receptors and transcriptomes reveal distinct immune niches and interactions in the adaptive immune response. Immunity.

[44] Dunn GP, Bruce AT, Ikeda H, Old LJ, Schreiber RD (2002). Cancer immunoediting: from immunosurveillance to tumor escape. Nat Immunol.

[45] Rosenthal R, Cadieux EL, Salgado R, Bakir MA, Moore DA, Hiley CT (2019). Neoantigen-directed immune escape in lung cancer evolution. Nature.

[46] McGranahan N, Furness AJ, Rosenthal R, Ramskov S, Lyngaa R, Saini SK (2016). Clonal neoantigens elicit T cell immunoreactivity and sensitivity to immune checkpoint blockade. Science.

[47] Joshi K, de Massy MR, Ismail M, Reading JL, Uddin I, Woolston A (2019). Spatial heterogeneity of the T cell receptor repertoire reflects the mutational landscape in lung cancer. Nat Med.

[48] Gerlinger M, Quezada SA, Peggs KS, Furness AJ, Fisher R, Marafioti T (2013). Ultra-deep T cell receptor sequencing reveals the complexity and intratumour heterogeneity of T cell clones in renal cell carcinomas. J Pathol.

[49] Reuben A, Gittelman R, Gao J, Zhang J, Yusko EC, Wu CJ (2017). TCR Repertoire Intratumor Heterogeneity in Localized Lung Adenocarcinomas: An Association with Predicted Neoantigen Heterogeneity and Postsurgical Recurrence. Cancer Discov.

[50] Wang Y, Swiecki M, Cella M, Alber G, Schreiber RD, Gilfillan S (2012). Timing and magnitude of type I interferon responses by distinct sensors impact CD8 T cell exhaustion and chronic viral infection. Cell Host Microbe.

[51] Han Y, Liu X, Wang Y, Wu X, Guan Y, Li H (2015). Identification of characteristic TRB V usage in HBV-associated HCC by using differential expression profiling analysis. Oncoimmunology.

[52] Nikolich-Zugich J, Slifka MK, Messaoudi I (2004). The many important facets of T-cell repertoire diversity. Nat Rev Immunol.

[53] Galon J, Costes A, Sanchez-Cabo F, Kirilovsky A, Mlecnik B, Lagorce-Pagès C (2006). Type, density, and location of immune cells within human colorectal tumors predict clinical outcome. Science.

[54] Savas P, Virassamy B, Ye C, Salim A, Mintoff CP, Caramia F (2018). Single-cell profiling of breast cancer T cells reveals a tissue-resident memory subset associated with improved prognosis. Nat Med.

[55] Willhauck M, Scheibenbogen C, Pawlita M, Möhler T, Thiel E, Keilholz U (2003). Restricted T-cell receptor repertoire in melanoma metastases regressing after cytokine therapy. Cancer Res.

[56] Oliveira G, Stromhaug K, Klaeger S, Kula T, Frederick DT, Le PM (2021). Phenotype, specificity and avidity of antitumour CD8. Nature.

[57] Ochsenreither S, Fusi A, Wojtke S, Busse A, Nüssler NC, Thiel E (2010). Comparison of T-cell receptor repertoire restriction in blood and tumor tissue of colorectal cancer patients. J Transl Med.

[58] Sherwood AM, Emerson RO, Scherer D, Habermann N, Buck K, Staffa J (2013). Tumor-infiltrating lymphocytes in colorectal tumors display a diversity of T cell receptor sequences that differ from the T cells in adjacent mucosal tissue. Cancer Immunol Immunother.

[59] Guo L, Bi X, Li Y, Wen L, Zhang W, Jiang W (2020). Characteristics, dynamic changes, and prognostic significance of TCR repertoire profiling in patients with renal cell carcinoma. J Pathol.

[60] Emerson RO, Sherwood AM, Rieder MJ, Guenthoer J, Williamson DW, Carlson CS (2013). High-throughput sequencing of T-cell receptors reveals a homogeneous repertoire of tumour-infiltrating lymphocytes in ovarian cancer. J Pathol.

[61] Looi CK, Chung FF, Leong CO, Wong SF, Rosli R, Mai CW (2019). Therapeutic challenges and current immunomodulatory strategies in targeting the immunosuppressive pancreatic tumor microenvironment. J Exp Clin Cancer Res.

[62] Bai X, Zhang Q, Wu S, Zhang X, Wang M, He F (2015). Characteristics of Tumor Infiltrating Lymphocyte and Circulating Lymphocyte Repertoires in Pancreatic Cancer by the Sequencing of T Cell Receptors. Sci Rep.

[63] Cui C, Tian X, Wu J, Zhang C, Tan Q, Guan X (2019). T cell receptor β-chain repertoire analysis of tumor-infiltrating lymphocytes in pancreatic cancer. Cancer Sci.

[64] Zhang J, Fujimoto J, Yusko E, Zhang J, Vignali M, Song X (2016). Intra-tumor heterogeneity of T cell receptor repertoire in lung cancers and its association with tumor genomic profile. J Clin Oncol.

[65] Joshi K, Ismail M, Reading JL, Massy MRD, Uddin I, Jamal-Hanjani M (2018). Characterisation of the TCR repertoire in NSCLC to reveal the relationship between TCR heterogeneity and genetic heterogeneity that is influenced by mutational load and is associated with disease recurrence. J Clin Oncol.

[66] Beausang JF, Wheeler AJ, Chan NH, Hanft VR, Dirbas FM, Jeffrey SS (2017). T cell receptor sequencing of early-stage breast cancer tumors identifies altered clonal structure of the T cell repertoire. Proc Natl Acad Sci U S A.

[67] Bortone DS, Woodcock MG, Parker JS, Vincent BG (2021). Improved T-cell Receptor Diversity Estimates Associate with Survival and Response to Anti-PD-1 Therapy. Cancer Immunol Res.

[68] Rooney MS, Shukla SA, Wu CJ, Getz G, Hacohen N (2015). Molecular and genetic properties of tumors associated with local immune cytolytic activity. Cell.

[69] Li B, Li T, Pignon JC, Wang B, Wang J, Shukla SA (2016). Landscape of tumor-infiltrating T cell repertoire of human cancers. Nat Genet.

[70] Chowell D, Krishna S, Becker PD, Cocita C, Shu J, Tan X (2015). TCR contact residue hydrophobicity is a hallmark of immunogenic CD8+ T cell epitopes. Proc Natl Acad Sci U S A.

[71] Zhuo Y, Yang X, Shuai P, Yang L, Wen X, Zhong X (2022). Evaluation and comparison of adaptive immunity through analyzing the diversities and clonalities of T-cell receptor repertoires in the peripheral blood. Front Immunol.

[72] Palermo B, Del Bello D, Sottini A, Serana F, Ghidini C, Gualtieri N (2010). Dacarbazine treatment before peptide vaccination enlarges T-cell repertoire diversity of melan-a-specific, tumor-reactive CTL in melanoma patients. Cancer Res.

[73] Franzese O, Palermo B, Di Donna C, Sperduti I, Ferraresi V, Stabile H (2016). Polyfunctional Melan-A-specific tumor-reactive CD8(+) T cells elicited by dacarbazine treatment before peptide-vaccination depends on AKT activation sustained by ICOS. Oncoimmunology.

[74] Palermo B, Franzese O, Donna CD, Panetta M, Quintarelli C, Sperduti I (2018). Antigen-specificity and DTIC before peptide-vaccination differently shape immune-checkpoint expression pattern, anti-tumor functionality and TCR repertoire in melanoma patients. Oncoimmunology.

[75] Sharma RA, McLelland HR, Hill KA, Ireson CR, Euden SA, Manson MM (2001). Pharmacodynamic and pharmacokinetic study of oral Curcuma extract in patients with colorectal cancer. Clin Cancer Res.

[76] Stuge TB, Holmes SP, Saharan S, Tuettenberg A, Roederer M, Weber JS (2004). Diversity and recognition efficiency of T cell responses to cancer. PLoS Med.

[77] Connerotte T, Van Pel A, Godelaine D, Tartour E, Schuler-Thurner B, Lucas S (2008). Functions of Anti-MAGE T-cells induced in melanoma patients under different vaccination modalities. Cancer Res.

[78] Wieckowski S, Baumgaertner P, Corthesy P, Voelter V, Romero P, Speiser DE (2009). Fine structural variations of alphabetaTCRs selected by vaccination with natural versus altered self-antigen in melanoma patients. J Immunol.

[79] Poran A, Scherer J, Bushway ME, Besada R, Balogh KN, Wanamaker A (2020). Combined TCR Repertoire Profiles and Blood Cell Phenotypes Predict Melanoma Patient Response to Personalized Neoantigen Therapy plus Anti-PD-1. Cell Rep Med.

[80] Ott PA, Hu-Lieskovan S, Chmielowski B, Govindan R, Naing A, Bhardwaj N (2020). A Phase Ib Trial of Personalized Neoantigen Therapy Plus Anti-PD-1 in Patients with Advanced Melanoma, Non-small Cell Lung Cancer, or Bladder Cancer. Cell.

[81] Fang H, Yamaguchi R, Liu X, Daigo Y, Yew PY, Tanikawa C (2014). Quantitative T cell repertoire analysis by deep cDNA sequencing of T cell receptor α and β chains using next-generation sequencing (NGS). Oncoimmunology.

[82] Tamura K, Hazama S, Yamaguchi R, Imoto S, Takenouchi H, Inoue Y (2016). Characterization of the T cell repertoire by deep T cell receptor sequencing in tissues and blood from patients with advanced colorectal cancer. Oncol Lett.

[83] D'Alise AM, Brasu N, De Intinis C, Leoni G, Russo V, Langone F (2022). Adenoviral-based vaccine promotes neoantigen-specific CD8. Sci Transl Med.

[84] Lang F, Schrörs B, Löwer M, Türeci Ö, Sahin U (2022). Identification of neoantigens for individualized therapeutic cancer vaccines. Nat Rev Drug Discov.

[85] Aurisicchio L, Pallocca M, Ciliberto G, Palombo F (2018). The perfect personalized cancer therapy: cancer vaccines against neoantigens. J Exp Clin Cancer Res.

[86] Brahmer J, Reckamp KL, Baas P, Crinò L, Eberhardt WE, Poddubskaya E (2015). Nivolumab versus Docetaxel in Advanced Squamous-Cell Non-Small-Cell Lung Cancer. N Engl J Med.

[87] Ferris RL, Blumenschein G, Fayette J, Guigay J, Colevas AD, Licitra L (2016). Nivolumab for Recurrent Squamous-Cell Carcinoma of the Head and Neck. N Engl J Med.

[88] Hodi FS, Hwu WJ, Kefford R, Weber JS, Daud A, Hamid O (2016). Evaluation of Immune-Related Response Criteria and RECIST v1.1 in Patients With Advanced Melanoma Treated With Pembrolizumab. J Clin Oncol.

[89] Motzer RJ, Escudier B, McDermott DF, George S, Hammers HJ, Srinivas S (2015). Nivolumab versus Everolimus in Advanced Renal-Cell Carcinoma. N Engl J Med.

[90] Robert C, Long GV, Brady B, Dutriaux C, Maio M, Mortier L (2015). Nivolumab in previously untreated melanoma without BRAF mutation. N Engl J Med.

[91] Lee JB, Kim HR, Ha SJ (2022). Immune Checkpoint Inhibitors in 10 Years: Contribution of Basic Research and Clinical Application in Cancer Immunotherapy. Immune Netw.

[92] Relecom A, Merhi M, Inchakalody V, Uddin S, Rinchai D, Bedognetti D (2021). Emerging dynamics pathways of response and resistance to PD-1 and CTLA-4 blockade: tackling uncertainty by confronting complexity. J Exp Clin Cancer Res.

[93] Hogan SA, Courtier A, Cheng PF, Jaberg-Bentele NF, Goldinger SM, Manuel M (2019). Peripheral Blood TCR Repertoire Profiling May Facilitate Patient Stratification for Immunotherapy against Melanoma. Cancer Immunol Res.

[94] Roh W, Chen PL, Reuben A, Spencer CN, Prieto PA, Miller JP (2017). Integrated molecular analysis of tumor biopsies on sequential CTLA-4 and PD-1 blockade reveals markers of response and resistance. Sci Transl Med.

[95] Robert L, Tsoi J, Wang X, Emerson R, Homet B, Chodon T (2014). CTLA4 blockade broadens the peripheral T-cell receptor repertoire. Clin Cancer Res.

[96] Gangaev A, Rozeman EA, Rohaan MW, Isaeva OI, Philips D, Patiwael S (2021). Differential effects of PD-1 and CTLA-4 blockade on the melanoma-reactive CD8 T cell response. Proc Natl Acad Sci U S A.

[97] Tumeh PC, Harview CL, Yearley JH, Shintaku IP, Taylor EJ, Robert L (2014). PD-1 blockade induces responses by inhibiting adaptive immune resistance. Nature.

[98] Kato T, Kiyotani K, Tomiyama E, Koh Y, Matsushita M, Hayashi Y (2021). Peripheral T cell receptor repertoire features predict durable responses to anti-PD-1 inhibitor monotherapy in advanced renal cell carcinoma. Oncoimmunology.

[99] Keenan TE, Burke KP, Van Allen EM (2019). Genomic correlates of response to immune checkpoint blockade. Nat Med.

[100] Valpione S, Galvani E, Tweedy J, Mundra PA, Banyard A, Middlehurst P (2020). Immune-awakening revealed by peripheral T cell dynamics after one cycle of immunotherapy. Nat Cancer.

[101] Fairfax BP, Taylor CA, Watson RA, Nassiri I, Danielli S, Fang H (2020). Peripheral CD8. Nat Med.

[102] Philip H, Snir T, Gordin M, Shugay M, Zilberberg A, Efroni S (2021). A T cell repertoire timestamp is at the core of responsiveness to CTLA-4 blockade. iScience.

[103] Luoma AM, Suo S, Wang Y, Gunasti L, Porter CBM, Nabilsi N (2022). Tissue-resident memory and circulating T cells are early responders to pre-surgical cancer immunotherapy. Cell.

[104] Oh DY, Cham J, Zhang L, Fong G, Kwek SS, Klinger M (2017). Immune Toxicities Elicted by CTLA-4 Blockade in Cancer Patients Are Associated with Early Diversification of the T-cell Repertoire. Cancer Res.

[105] Salih Z, Banyard A, Tweedy J, Galvani E, Middlehurst P, Mills S (2022). T cell immune awakening in response to immunotherapy is age-dependent. Eur J Cancer.

[106] Aoki H, Shichino S, Matsushima K, Ueha S (2022). Revealing Clonal Responses of Tumor-Reactive T-Cells Through T Cell Receptor Repertoire Analysis. Front Immunol.

[107] Lucca LE, Axisa PP, Lu B, Harnett B, Jessel S, Zhang L (2021). Circulating clonally expanded T cells reflect functions of tumor-infiltrating T cells. J Exp Med.

[108] Pauken KE, Shahid O, Lagattuta KA, Mahuron KM, Luber JM, Lowe MM (2021). Single-cell analyses identify circulating anti-tumor CD8 T cells and markers for their enrichment. J Exp Med.

[109] Provencio M, Nadal E, Insa A, García-Campelo MR, Casal-Rubio J, Dómine M (2020). Neoadjuvant chemotherapy and nivolumab in resectable non-small-cell lung cancer (NADIM): an open-label, multicentre, single-arm, phase 2 trial. Lancet Oncol.

[110] Provencio M, Serna-Blasco R, Nadal E, Insa A, García-Campelo MR, Casal Rubio J (2022). Overall Survival and Biomarker Analysis of Neoadjuvant Nivolumab Plus Chemotherapy in Operable Stage IIIA Non-Small-Cell Lung Cancer (NADIM phase II trial). J Clin Oncol.

[111] Casarrubios M, Cruz-Bermúdez A, Nadal E, Insa A, García Campelo MDR, Lázaro M (2021). Pretreatment Tissue TCR Repertoire Evenness Is Associated with Complete Pathologic Response in Patients with NSCLC Receiving Neoadjuvant Chemoimmunotherapy. Clin Cancer Res.

[112] Hui Z, Zhang J, Ren Y, Li X, Yan C, Yu W (2022). Single-cell profiling of immune cells after neoadjuvant pembrolizumab and chemotherapy in IIIA non-small cell lung cancer (NSCLC). Cell Death Dis.

[113] Han J, Duan J, Bai H, Wang Y, Wan R, Wang X (2020). TCR Repertoire Diversity of Peripheral PD-1. Cancer Immunol Res.

[114] Schmid P, Adams S, Rugo HS, Schneeweiss A, Barrios CH, Iwata H (2018). Atezolizumab and Nab-Paclitaxel in Advanced Triple-Negative Breast Cancer. N Engl J Med.

[115] Cortes J, Cescon DW, Rugo HS, Nowecki Z, Im SA, Yusof MM (2020). Pembrolizumab plus chemotherapy versus placebo plus chemotherapy for previously untreated locally recurrent inoperable or metastatic triple-negative breast cancer (KEYNOTE-355): a randomised, placebo-controlled, double-blind, phase 3 clinical trial. Lancet.

[116] Miles D, Gligorov J, André F, Cameron D, Schneeweiss A, Barrios C (2021). Primary results from IMpassion131, a double-blind, placebo-controlled, randomised phase III trial of first-line paclitaxel with or without atezolizumab for unresectable locally advanced/metastatic triple-negative breast cancer. Ann Oncol.

[117] Shah AN, Flaum L, Helenowski I, Santa-Maria CA, Jain S, Rademaker A (2020). Phase II study of pembrolizumab and capecitabine for triple negative and hormone receptor-positive, HER2-negative endocrine-refractory metastatic breast cancer. J Immunother Cancer.

[118] Chun B, Pucilowska J, Chang S, Kim I, Nikitin B, Koguchi Y (2022). Changes in T-cell subsets and clonal repertoire during chemoimmunotherapy with pembrolizumab and paclitaxel or capecitabine for metastatic triple-negative breast cancer. J Immunother Cancer.

[119] Zitvogel L, Apetoh L, Ghiringhelli F, Kroemer G (2008). Immunological aspects of cancer chemotherapy. Nat Rev Immunol.

[120] Nolan E, Savas P, Policheni AN, Darcy PK, Vaillant F, Mintoff CP (2017). Combined immune checkpoint blockade as a therapeutic strategy for *BRCA1*-mutated breast cancer. Sci Transl Med.

[121] Franzese O, Battaini F, Graziani G, Tentori L, Barbaccia ML, Aquino A (2018). Drug-induced xenogenization of tumors: a possible role in the immune control of malignant cell growth in the brain?. Pharmacol Res.

[122] Crisafulli G, Sartore-Bianchi A, Lazzari L, Pietrantonio F, Amatu A, Macagno M (2022). Temozolomide Treatment Alters Mismatch Repair and Boosts Mutational Burden in Tumor and Blood of Colorectal Cancer Patients. Cancer Discov.

[123] Shugay M, Britanova OV, Merzlyak EM, Turchaninova MA, Mamedov IZ, Tuganbaev TR (2014). Towards error-free profiling of immune repertoires. Nat Methods.

[124] Rosati E, Dowds CM, Liaskou E, Henriksen EKK, Karlsen TH, Franke A (2017). Overview of methodologies for T-cell receptor repertoire analysis. BMC Biotechnol.

[125] Macosko EZ, Basu A, Satija R, Nemesh J, Shekhar K, Goldman M (2015). Highly Parallel Genome-wide Expression Profiling of Individual Cells Using Nanoliter Droplets. Cell.

[126] Goldstein LD, Chen YJ, Dunne J, Mir A, Hubschle H, Guillory J (2017). Massively parallel nanowell-based single-cell gene expression profiling. BMC Genomics.

[127] Redmond D, Poran A, Elemento O (2016). Single-cell TCRseq: paired recovery of entire T-cell alpha and beta chain transcripts in T-cell receptors from single-cell RNAseq. Genome Med.

[128] Weber K, Bartsch U, Stocking C, Fehse B (2008). A multicolor panel of novel lentiviral "gene ontology" (LeGO) vectors for functional gene analysis. Mol Ther.

[129] Goh JJL, Chou N, Seow WY, Ha N, Cheng CPP, Chang YC (2020). Highly specific multiplexed RNA imaging in tissues with split-FISH. Nat Methods.

[130] Hudson WH, Sudmeier LJ (2022). Localization of T cell clonotypes using the Visium spatial transcriptomics platform. STAR Protoc.

[131] Bolotin DA, Poslavsky S, Mitrophanov I, Shugay M, Mamedov IZ, Putintseva EV (2015). MiXCR: software for comprehensive adaptive immunity profiling. Nat Methods.

[132] Kuchenbecker L, Nienen M, Hecht J, Neumann AU, Babel N, Reinert K (2015). IMSEQ–a fast and error aware approach to immunogenetic sequence analysis. Bioinformatics.

[133] Li S, Lefranc MP, Miles JJ, Alamyar E, Giudicelli V, Duroux P (2013). IMGT/HighV QUEST paradigm for T cell receptor IMGT clonotype diversity and next generation repertoire immunoprofiling. Nat Commun.

[134] Gerritsen B, Pandit A, Andeweg AC, de Boer RJ (2016). RTCR: a pipeline for complete and accurate recovery of T cell repertoires from high throughput sequencing data. Bioinformatics.

[135] Ye J, Ma N, Madden TL, Ostell JM (2013). IgBLAST: an immunoglobulin variable domain sequence analysis tool. Nucleic Acids Res.

[136] Morin A, Kwan T, Ge B, Letourneau L, Ban M, Tandre K (2016). Immunoseq: the identification of functionally relevant variants through targeted capture and sequencing of active regulatory regions in human immune cells. BMC Med Genomics.

[137] Munshaw S, Kepler TB (2010). SoDA2: a Hidden Markov Model approach for identification of immunoglobulin rearrangements. Bioinformatics.

[138] Gaëta BA, Malming HR, Jackson KJ, Bain ME, Wilson P, Collins AM (2007). iHMMune-align: hidden Markov model-based alignment and identification of germline genes in rearranged immunoglobulin gene sequences. Bioinformatics.

[139] Vander Heiden JA, Yaari G, Uduman M, Stern JN, O'Connor KC, Hafler DA (2014). pRESTO: a toolkit for processing high-throughput sequencing raw reads of lymphocyte receptor repertoires. Bioinformatics.

[140] Stubbington MJT, Lönnberg T, Proserpio V, Clare S, Speak AO, Dougan G (2016). T cell fate and clonality inference from single-cell transcriptomes. Nat Methods.

[141] Rizzetto S, Koppstein DNP, Samir J, Singh M, Reed JH, Cai CH (2018). B-cell receptor reconstruction from single-cell RNA-seq with VDJPuzzle. Bioinformatics.

[142] Zhang Z, Xiong D, Wang X, Liu H, Wang T (2021). Mapping the functional landscape of T cell receptor repertoires by single-T cell transcriptomics. Nat Methods.

[143] Marrero I, Aguilera C, Hamm DE, Quinn A, Kumar V (2016). High-throughput sequencing reveals restricted TCR Vβ usage and public TCRβ clonotypes among pancreatic lymph node memory CD4(+) T cells and their involvement in autoimmune diabetes. Mol Immunol.

[144] Zhao Y, Nguyen P, Vogel P, Li B, Jones LL, Geiger TL (2016). Autoimmune susceptibility imposed by public TCRβ chains. Sci Rep.

[145] Shugay M, Bagaev DV, Turchaninova MA, Bolotin DA, Britanova OV, Putintseva EV (2015). VDJtools: Unifying Post-analysis of T Cell Receptor Repertoires. PLoS Comput Biol.

[146] DeWitt WS, Emerson RO, Lindau P, Vignali M, Snyder TM, Desmarais C (2015). Dynamics of the cytotoxic T cell response to a model of acute viral infection. J Virol.

[147] Hosoi A, Takeda K, Nagaoka K, Iino T, Matsushita H, Ueha S (2018). Increased diversity with reduced "diversity evenness" of tumor infiltrating T-cells for the successful cancer immunotherapy. Sci Rep.

[148] Zhigalova EA, Izosimova AI, Yuzhakova DV, Volchkova LN, Shagina IA, Turchaninova MA (2020). RNA-Seq-Based TCR Profiling Reveals Persistently Increased Intratumoral Clonality in Responders to Anti-PD-1 Therapy. Front Oncol.

[149] Rousseau R, Van Hecke P, Nijssen D, Bogaert J. The relationship between diversity profiles, evenness and species richness based on partial ordering. Economic J. 1999;348–61.

[150] Simpson EH. Measurement of diversity. Nature. 1949;163:688.

[151] Jaccard P. The distribution of the flora in the alpine zone. New Phytologist. 1912;11:37–50.

[152] Rempala GA, Seweryn M (2013). Methods for diversity and overlap analysis in T-cell receptor populations. J Math Biol.

[153] Pielou EC. The measurement of diversity in different types of biological collections. J Theor Biol. 1966;13:131–44.

[154] Shannon CE. A mathematical theory of communication. Bell Syst Tech J. 1948;27:379–423, 623–56.

